# Chemical Characterization and Effect of a Lactobacilli-Postbiotic on *Streptococcus mutans* Biofilm In Vitro

**DOI:** 10.3390/microorganisms12050843

**Published:** 2024-04-23

**Authors:** Guilherme Bandeira Santana, Patrick Veras Quelemes, Enedina Rodrigues da Silva Neta, Sidney Gonçalo de Lima, Gláuber Campos Vale

**Affiliations:** 1 Postgraduate Program in Dentistry, Federal University of Piauí, Teresina 64049-550, Brazil; guilhermesantanaodonto@gmail.com (G.B.S.); pquelemes@gmail.com (P.V.Q.); 2 Organic Geochemistry Laboratory, Postgraduate Program in Chemistry, Federal University of Piauí, Teresina 64049-550, Brazil; enedinarodriguees@hotmail.com (E.R.d.S.N.); sidney@ufpi.edu.br (S.G.d.L.)

**Keywords:** antimicrobial agents, dental caries, oral microbiome, chemical profile, dentistry

## Abstract

Postbiotic is the term used to define the soluble factors, metabolic products, or byproducts released by live probiotic bacteria or after its lysis. The objective of this study was to carry out the chemical characterization of the postbiotic of *Lacticaseibacillus rhamnosus* LR-32 and to evaluate its in vitro effect on the development of the *Streptococcus mutans* biofilm. After the cultivation of the probiotic strain, the postbiotic was extracted by centrifuging the culture and filtering the supernatant. This postbiotic was characterized by using gas chromatography coupled with mass spectrometry (GC–MS), and then it was used to determine the growth inhibition of *S. mutans* in its planktonic form; additionally, its effects on the following parameters in 48 h biofilm were evaluated: viable bacteria, dry weight, and gene expression of glucosyltransferases and VicR gene. The control group consisted of the biofilm without any treatment. A paired *t*-test was performed for statistical analysis, with the *p*-value set at 5%. Seventeen compounds of various chemical classes were identified in the postbiotic, including sugars, amino acids, vitamins, and acids. The treatment with the postbiotic led to an inhibition of the growth of *S. mutans* in its planktonic form, as well as a decrease in the number of viable bacteria, reduction in dry weight, and a negative regulation of the gene expression of gtfB, gtfC, gtfD, and vicR in its biofilm state, compared with the nontreated group (*p* < 0.05). The postbiotic of *L. rhamnosus* impaired the development of *S. mutans* biofilm.

## 1. Introduction

Probiotics are live microorganisms that, in ideal quantities, can provide health benefits due to their antibacterial, protective, nutritional, and immunomodulatory functions [1]. *Lacticaseibacillus rhamnosus*, a facultative anaerobic and Gram-positive probiotic bacterium [2], has been frequently and effectively studied in the prevention and treatment of various gastrointestinal diseases [3]. Specifically, the *L. rhamnosus* LR-32 strain is considered safe for human consumption and has shown an in vitro inhibitory effect against pathogens such as *Staphylococcus aureus* and *Escherichia coli*, being able to modulate immune function [4]. Furthermore, *L. rhamnosus* Lr-32 was able to reduce *H. pylori* adhesion and inflammation caused by *H. pylori* infection in human gastric adenocarcinoma cells [5].

Several studies presented positive results regarding oral health after the addition of probiotics to treat periodontal diseases [6,7,8,9,10] in addition to reducing plaque index and gingival index [11]. The positive effect of probiotics on *Streptococcus mutans*, one of the main microorganisms involved in the development of dental caries, is also proven both for the reduction and inhibition of these bacteria [12]. Furthermore, *L. rhamnosus* LR-32 presented an effect on the control of candidiasis, since its incorporation as an additive in cheese decreased the levels of *Candida albicans* colonization in denture wearers [13]. Also, *Lactobacillus* supernatant exhibited effects against *Candida*/bacteria adhesion and biofilm formation on silicone [14].

In turn, postbiotic is the term used to define soluble factors, products, or metabolic byproducts released by live bacteria or released after bacterial lysis [15,16]. Such products are excreted into the cell-free supernatant of the bacterial suspension during its growth and can provide health benefits through a direct or indirect mechanism [17]. Postbiotics have an advantage over probiotics since they optimize their beneficial effects, bypass the technical challenge of colonization efficiency, and keep microorganisms viable and stable in the product at high doses, since they are influenced by external factors, such as temperature and pH [18]. Additionally, postbiotics can be added to toothpaste, chewing gum, natto, chips, popcorn, and lollipops [19].

Lactobacillus-derived postbiotics have been shown to inhibit biofilms of several organisms, including pathogens such as *C. albicans* [20], *S. aureus* [21], and *Porphyromonas gingivalis* [22]. This effect may be related to the chemical composition of the postbiotic used [18]. Particularly, the postbiotic strain *L. rhamsnosus* LR-32 had a positive effect on periodontopathogenic bacteria, reducing the expression of inflammatory genes in gingival epithelial cells, and presented potential for antibacterial activity in periodontal tissues [10]. However, there are no studies showing the chemical composition and effect of this strain of postbiotic for *S. mutans* bacteria and biofilms.

Thus, this work aimed to carry out the chemical characterization of the postbiotic *L. rhamnosus* LR-32 and evaluate its effect on the development of the *S. mutans* biofilm.

## 2. Materials and Methods

### 2.1. Obtaining the Postbiotic (Conditioned Culture Medium)

The postbiotic (conditioned culture medium) was obtained from the probiotic strain *L. rhamnosus* LR-32 (Danisco, Madison, WI, USA) as previously described [10]. Briefly, the strain was initially cultivated using MRS Lactobacilli agar in 5% CO_2_ (microaerophilic condition) at 37 °C for 48 h. The colonies were cultured in MRS broth at 37 °C for 18 h and then centrifuged at 5000× *g* for 10 min at 4 °C. The supernatant was transferred to a new tube and filtered through a polyvinylidene fluoride (PVDF) filter with a pore size of 0.22 μm (Millipore Co., Billerica, MA, USA).

The postbiotic was cultivated in brain heart infusion (BHI) agar to prove its sterility. The product was packaged in a 2 mL tube and frozen in a −20 °C freezer for the antimicrobial tests.

Subsequently, the obtained postbiotic was freeze-dried using a benchtop freeze dryer for 48 h (LS300; Terroni, São Paulo, Brazil), with freezing temperature: −40 °C, pump pressure: 100 mTorr and shelf temperature: −60 °C. The samples were then placed in a desiccator containing phosphorus pentoxide for desiccation and subsequent chemical analysis.

### 2.2. Gas Chromatography Coupled with Mass Spectrometry (GC–MS)

#### 2.2.1. Analysis Method

Gas chromatography coupled with mass spectrometry (GC–MS) analysis was con-ducted using a chromatograph (GC–MS-QP2010 SE, AOC-5000 autoinjector from SHIMADZU) under the following conditions: injector temperature set at 290 °C, initial oven temperature of 60 °C, with a heating ramp of 4 °C/min up to 315 °C, maintaining this temperature for 25 min, a split ratio of 1:10, interface temperature of 320 °C, and source temperature of 230 °C. The mass range was from 57 to 700 Daltons, with electron impact ionization set at 70 eV. A DB5-MS UI column (Agilent, Santa Clara, CA, USA), measuring 30 m × 0.25 mm and featuring an internal film thickness of 0.25 µm, was utilized for chromatographic separation of components. The stationary phase consisted of diphenyl dimethylpolysiloxane, and helium was the carrier gas. Identification and quantification of the constituents were achieved by comparing retention indices, interpreting fragmentation patterns observed in the mass spectra, cross-referencing with the analysis system’s database (considering only spectra with a similarity index equal to or greater than 90%), and consulting relevant data from the literature (Wiley Registry/NIST Mass Spectral Library 2023).

#### 2.2.2. Silylation Method

Before the derivatization process, the samples were placed in a container containing phosphorus pentoxide for 24 h. Approximately 5.0 mg of sample was used in a 5 mL round-bottomed flask, cleaned with dichloromethane, containing a magnetic bar for derivatization. Then, with the aid of a glass syringe, pyridine and BSTFA (N,O-bis(trimethylsilyl)trifluoroacetamide) were added in a 1:1 (*v*/*v*) ratio, and the acidic fraction was added, obeying the proportion of 1 mg of fraction for each 50 µL of BSTFA and 50 µL of pyridine. The derivatization flask was placed in a sand bath system coupled with a thermometer under a heating plate, maintaining the temperature between 50 °C and 70 °C for 1 h and 30 min. After silylation, the samples were filtered directly into the injection vial with a PTFE syringe filter (0.22 µm, 13 mm) (Allcrom, São Paulo, Brazil). After filtration, the volume was made up to 1.0 mL with ethyl acetate.

### 2.3. Effect on Planktonic Cells of S. mutans

Aliquots (250 µL) of *L. rhamnosus* LR-32 conditioned medium (prepared as described in item 2.1) or PBS and 250 µL of 10^8^ CFU/mL of *S. mutans* UA130 (ATCC 700611) were inoculated into 5 mL of BHI broth with 0.12% glucose. Then, bacterial growth was monitored at time zero and in regular intervals within 24 h, in triplicate, determining the optical density at 600 nm in a spectrophotometer.

### 2.4. Effect on Biofilm of S. mutans

*S. mutans* UA130 was cultured in tubes containing BHI broth plus 1% sucrose for 18 h. After this period, the suspension was vortexed, and 2 mL (approximately 1 × 10^6^ CFU/mL) was dispensed in 24-well plates on hydroxyapatite (HA) disks (2.93 cm^2^; Clarkson Chromatography Products, Inc., South Williamsport, PA, USA) for 8 h, for initial bacterial adhesion. Previously, the disks were incubated with clarified saliva and filtered on a 0.22 μm polyvinylidene fluoride (PVDF) filter for 1 h at 37 °C in 24-well plates.

Then, the discs were gently washed with sterile 0.9% NaCl saline solution and the culture medium BHI broth plus 1% sucrose, containing the conditioned medium of *L. rhamnosus* LR-32 (prepared as described in item 2.1) at a dilution of 1:4 (culture medium: conditioned medium), was added to the *S. mutans* biofilms adhered to the discs. Untreated biofilms were considered as the control group. The plates were incubated in microaerophilic condition at 37 °C for 24 h.

At the end of the experiment, the biofilms were gently washed with saline solution, and collected to determine viable bacteria, dry weight, and gene expression by RT-qPCR. The experiment was carried out in triplicate, and within each experiment, four biofilm samples were used for each of the analyses, described as follows.

#### 2.4.1. Determination of Live Bacteria Count

*S. mutans* biofilm samples were added with saline solution, at a proportion of 1 mL/mg of wet weight, homogenized in a vortex tube shaker and sonicated at 40 W, 5% amplitude, 6 pulses of 10 s each. From this suspension, dilutions with saline solution were made in the proportion of 10^−1^ to 10^−5^ times and inoculated on the BHI agar, to count total bacteria. Colony-forming units (CFUs) were counted, and the results were expressed as CFU/mg of wet biofilm.

#### 2.4.2. Determination of Dry Weight of Biofilms

Biofilm samples were dried in a desiccator containing phosphorus pentoxide for 24 h, and the dry weight of the biofilm was determined in microtubes previously weighed on a precision analytical balance.

#### 2.4.3. Effect of Postbiotics on Gene Expression of *S. mutans*

Glucan production by *S. mutans* was analyzed indirectly using gtfs expression comparison. Furthermore, the expression of the vicR gene (vic, for virulence control) was determined. Biofilms were washed three times with PBS. Total RNA was then isolated with a TRIzol^®^ Max Bacterial RNA Isolation Kit (Invitrogen Life Tech, Carlsbad, CA, USA) according to the manufacturer’s instructions. The cDNA was synthesized by mixing total RNA (1 mg) and Maxime™ RT Premix (random primer; INTON, Gyeonggi, Republic of Korea) in a 20 μL reaction volume and by incubating the mixture at 45 °C for 1 h. The cDNAs were mixed with 10 μL of SYBR PremixExTaq and 0.4 μM of each primer pair in 20 μL final volume and subjected to 40 cycles of PCR (95 °C for 15 s, 60 °C for 15 s, and 72 °C for 33 s) with real-time PCR model StepOne Plus (Thermo Fisher, Foster City, CA, USA). PCR products were investigated for each amplification product using an amplification dissociation curve. The 16S rRNA gene (housekeeping gene) was used as a reference to normalize expression levels and to quantify changes in gtfs and VicR expression between samples treated with postbiotics and untreated samples. The primer sequences for real-time RT-PCR are described in Table 1.

### 2.5. Statistical Analysis

Experiments were carried out in triplicate with four replications within each experiment. The data generated were analyzed by one-way analysis of variance (ANOVA) and Tukey’s post hoc test using the Graphpad Prism 9.02 program (Graphpad, La Jolla, CA, USA). The results were considered significant for *p*-values lower than 0.05.

## 3. Results

### 3.1. Chemical Characterization

**Figure 1:**
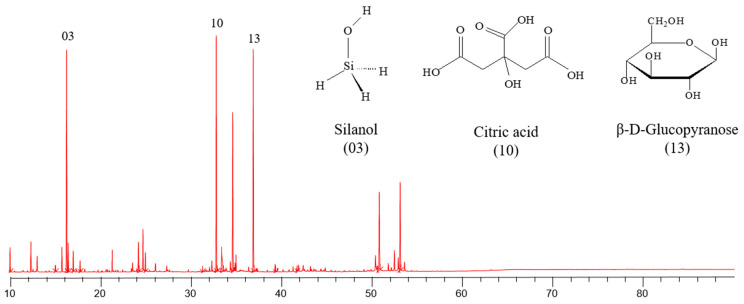
Chromatographic profile of *L. rhamnosus* LR-32 postbiotic that was silylated in BSTFA and analyzed by GC–MS.

The chromatographic profile of the *L. rhamnosus* LR-32 postbiotic is presented in Figure 1. The majority of species in the sample are silanol (03), citric acid (10), and β-D-glucopyranose (13), thus exerting a greater influence on the characteristics of the postbiotic. In addition to these, other compounds present were α-D-galactopyranose, lactose, and maltose, showing the great abundance of sugars in the sample.

Table 2 presents the chemical composition of *L. rhamnosus* LR-32 postbiotic, silylated in BSTFA and analyzed by using GC–MS. In the chromatogram, a total of 17 (seventeen) compounds were identified, from various chemical classes, including 5 (five) sugars, 2 (two) amino acids, 1 (one) vitamin, and 3 (three) acids, among others.

### 3.2. Effect of the Postbiotics on S. mutans

**Figure 2:**
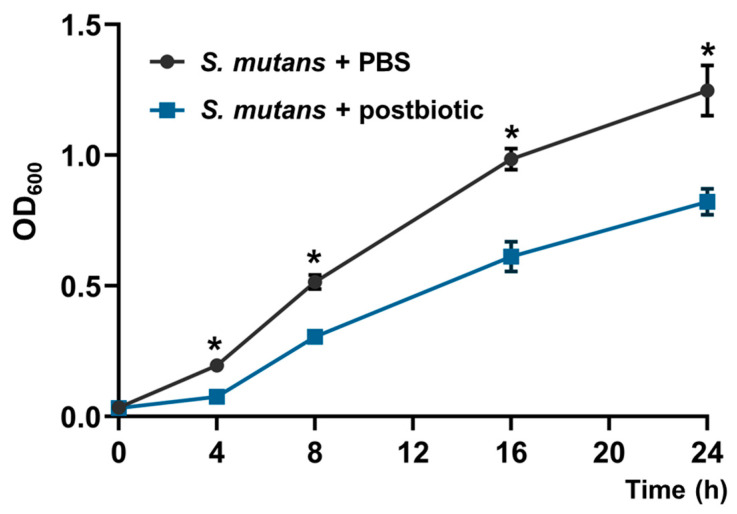
Effect of *L. rhamnosus* LR-32 postbiotic on the growth of *S. mutans* determined by optical density (OD at 600 nm); * *p* < 0.05.

Figure 2 shows the effect of *L. rhamnosus* LR-32 postbiotic on the growth of *S. mutans* determined by optical density (600 nm) over 24 h. When analyzing the growth of planktonic bacteria, an attenuation was observed in comparison to the control group containing PBS, showing statistical differences in the periods of 4 h, 8 h, 16 h, and 24 h.

**Figure 3:**
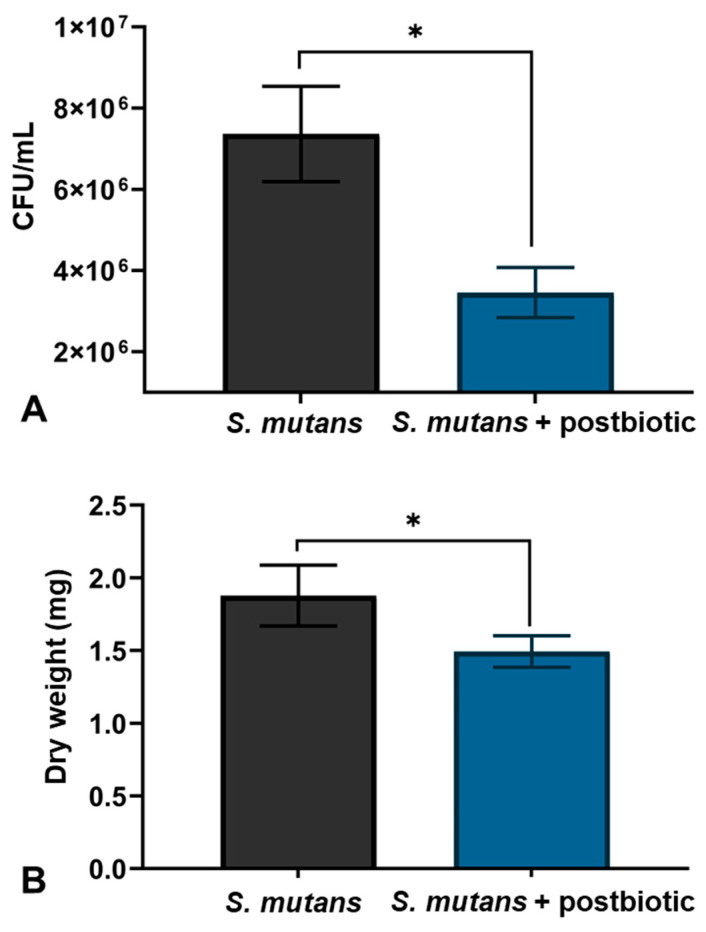
(**A**) Count of viable bacteria in *S. mutans* biofilms (CFU/mL) formed with and without the postbiotic *L. rhamnosus* LR-32. (**B**) Dry weight of *S. mutans* biofilms formed (mg) with and without the postbiotic *L. rhamnosus* LR-32. The vertical bars indicate the standard deviation; * *p* < 0.05.

Figure 3A shows the count of viable bacteria in *S. mutans* biofilms formed over 48 h (CFU/mL). There was less bacterial colonization in formed biofilms in which the postbiotic was added, showing a significant difference when compared to the control group (untreated biofilms).

Figure 3B shows the dry weight of *S. mutans* biofilms formed over 48 h of treatment and after being dried for 24 h in a desiccator with phosphorus pentoxide (mg). The dry weight of the biofilms that were treated with the conditioned medium was lower, with a statistically significant difference in comparison to the control group.

**Figure 4:**
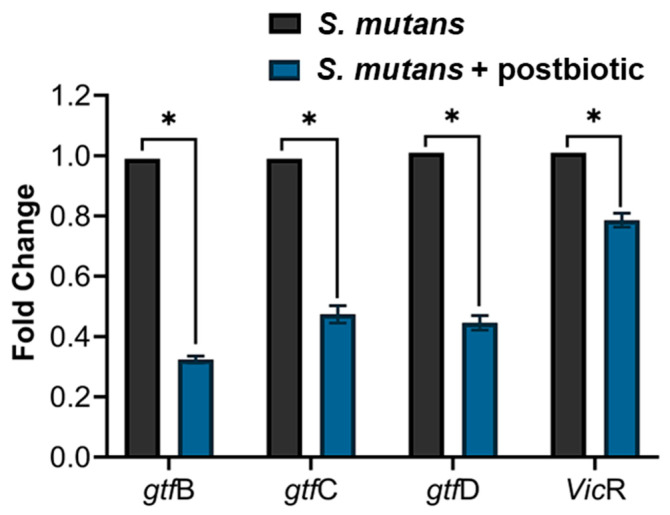
Relative gene expression (mean ± SD of foldchange) of the gtfB, gtfC, gtfD, and VicR genes of *S. mutans* with and without treatment with the postbiotic of *L. rhamnosus* LR-32 by RT-PCR; * *p* < 0.05.

Figure 4 shows the relative gene expression (mean ± SD of foldchange) of the gtfB, gtfC, gtfD, and VicR genes of *S. mutans* by RT-PCR. Lower gene expression can be observed in all genes analyzed with a statistically significant difference in the groups treated with the postbiotic when compared to the control group.

## 4. Discussion

This study determined the composition of a postbiotic derived from *L. rhamnosus* LR-32 and its effect on the development of *S. mutans* biofilms in vitro. The results indicate a reduction in the development of these biofilms by analyzing the following parameters: viability, dry weight, and expression of genes associated with the virulence of that cariogenic bacterium. This result may be due to the presence of exo-metabolites in the analyzed postbiotic, such as acids and antimicrobial products like hydrogen peroxide [25]. When observing the majority of compounds (Table 2), there is a certain abundance of organic acids, peptides, and amino acids, corroborating the studies by Hossain et al. [26] and Moradi et al. [18], where the same classes of compounds were found.

It is important to mention that there may still be other types of compounds; furthermore, the loss or modification of metabolites when going through purification and/or detection processes cannot be ruled out [26]. It is known that *L. rhamnosus* LR-32 can produce hydrogen peroxide [22]; however, in the freeze-drying process of the conditioned medium, materials such as hydrogen peroxide and oxygen metabolites lose their antimicrobial activity [27]. When comparing the chemical profile of the postbiotic studied with other postbiotics like the ones derived from *Lactobacillus* spp., a diversity of profiles is observed, which can be explained by the type of bacteria, the analysis methods (GC–MS or HPLC), and the nature of the conditioned medium [18].

In the present study, the presence of Silanol (17.98%) in the postbiotic was observed, which may be related to the reduction in CFU of *S. mutans* (Figure 3), since, similarly, a reduction in CFUs of *E. coli* (Gram-negative) and *S. aureus* (Gram-positive) was observed in a study that analyzed the surface potential of biomaterials with silanol groups to prevent the formation of bacterial biofilm [28]. Furthermore, regarding biofilm formation prevention, that study demonstrated that silanol on the surface of the biomaterial showed an effect only on the *S. aureus* biofilm, suggesting an effect only on Gram-positive bacteria, such as *S. mutans*.

The presence of citric acid, another abundant compound in the postbiotic evaluated, may also be related to the reduction in the number of viable bacteria and is considered a safe disinfectant. A study conducted evaluated the effectiveness of citric acid and sodium hypochlorite as disinfectants against the bacteria *Mycoplasma bovis*; this study demonstrated that a concentration of 0.5% citric acid significantly reduces the infectivity of such bacteria [29]. Moreover, the antimicrobial effect of using citric acid, which occurs through membrane damage and loss of viability, at concentrations of 1% and 10% at pH 9.5 on *E. coli* and *K. aerogenes* was shown in another study [30].

The decreased growth of *S. mutans* in this study may also have been caused by organic acids produced by *L. rhamnosus* present in the conditioned medium. Lactic acid bacteria can produce a series of growth inhibitors, such as organic acids, bacteriocins, and hydrogen peroxide, which have an effect mainly against Gram-positive pathogens [31]. Additionally, probiotic bacteria can inhibit the growth of *S. mutans*, especially when looking at the effect of *Lactobacillus paracasei*, *L. plantarum*, and *L. rhamnosus,* which present the best results against this microorganism [32]. It was also observed that culture supernatants of *L. rhamnosus* GG, *L. plantarum* 299V, *L. reuteri* PTA 5289, and SD2112 reduced the viability of *S. mutans* [33], and the antimicrobial activity depended on the acidity level of the lactobacilli culture, which may explain the results of our study since the postbiotic used was not neutralized.

The dry weight of biofilms was reduced when exposed to the postbiotic, which can be highlighted by the lower number of viable bacteria, but also by the decrease in the polysaccharide matrix of the *S. mutans* biofilm. That matrix is composed of extracellular polysaccharides that are produced from the fragmentation of the sucrose molecule by enzymes—glucosyltransferases—produced by the cariogenic bacteria. These enzymes are responsible for the production of glucans from the glucose molecule originating from sucrose, which is responsible for producing acids and making the biofilm more cariogenic [34]. The decrease observed in the polysaccharide matrix, resulting from the decrease in the production of insoluble glucans due to the conditioned medium, was previously described [23] and corroborates with decreases observed in the present study.

The ability of *S. mutans* to produce extracellular glucans from sucrose using glucosyltransferases (gtfs) is considered a virulence factor of this bacterium [35]. For that species, there are three well-described glucosyltransferases. The *gtf*B gene product provides cellular aggregation and cohesion of different bacterial strains, enabling bacteria to produce glucans. The *gtf*C is the gene most related to the acquired pellicle and is the one with the greatest capacity for adhesion to hydroxyapatite among the three gtfs. Glucosyltransferase-D generates water-soluble glucans that can be metabolized or stored by bacterial strains. In addition to these, the *vicRK* gene exerts a self-protective function in response to external stress, being related to the signal transduction system; this gene also modulates gene expression in response to acid tolerance and oxidative stress [24].

As shown in Figure 4, a lower gene expression can be observed in all analyzed genes (*gtf*B, *gtf*C, *gtf*D, and *vic*R) when RT-PCR was performed, showing a negative regulation when compared to the control group. A similar result was observed in a study where the biosurfactant produced by the analyzed postbiotic significantly decreased the gene expression of gtfB and gtfC in *S. mutans* biofilms [35]. Similarly, bacterial lysates (BLs), another type of postbiotic, of *Lactobacillus plantarum* and *L. rhamnosus* GG downregulated *gtf*B, *gtf*C, and *gtf*D by *S. mutans*, suggesting that BLs reduce the synthesis of extracellular polysaccharide and thereby reduce *S. mutans* biofilm, but did not eradicate the established *S. mutans* biofilm [36].

This research has the limitations of an in vitro study, and the results must be interpreted with caution since the biofilm formed in the oral cavity is much more complex than the single-specie model used in the present research. Moreover, although postbiotics are considered superior to probiotics because of their good acid–base and thermal stability, their ease of storage and use, and high safety [19], further research should be carried out with other types of postbiotics (peptidoglycans, lipopolysaccharides, and pili) to test the safety and effectiveness of these formulations in the oral cavity.

## 5. Conclusions

The postbiotic from *L. rhamnosus* LR-32, characterized by 17 diverse chemical compounds, primarily citric acid, silanol, and β-D-glucopyranose, exhibited notable inhibitory effects on *S. mutans* growth in both planktonic and biofilm states in vitro. This intervention reduced biofilm dry weight and downregulated essential genes (*gtf*B, *gtf*C, *gtf*D, *vic*R). These findings underscore the postbiotic’s potential in countering *S. mutans* and its biofilm development, offering promising implications for future oral health strategies of antimicrobial management.

## Figures and Tables

**Table 1:** Sequences of the primers used in the study.

Gene	Description	Primer Sequence (5′-3′)	Reference
*gtf*B	Production of water-insoluble glucan	5′-AGC AAT GCA GCC AAT CTA CAA AT-3′ 5′-ACG AAC TTT GCC GTT ATT GTC A-3′	[23]
*gtf*C	Production of water-soluble and insoluble glucan	5′-CTC AAC CAA CCG CCA CTG TT-3′ 5′-GGT TAA CGT CAA TAG AAT CTG TAG TAG C-3′	[23]
*gtf*D	Production of water-soluble glucan	5′-CAC AGG CAA AAG CTG AAT TAC A-3′ 5′-GAT GGC CGC TAA GTC AAC AG-3′	[23]
*vic*R	Response Regulator	5′-TGA CACGA TTA CAG CCT TTG ATG-3′ 5′-CGT CTA GTT CTG GTA ACA TTA AGT CCA ATA-3′	[24]
16S rRNA	Internal standard normalizer	5′-AGT CTG AGT 5′-GAA TAA AAG GCT A-3′ 5′-GTT AGC TCC GGC ACT AAG CC-3′	[23]

**Table 2:** Chemical composition of *L. rhamnosus* LR-32 postbiotic that was silylated in BSTFA and analyzed by GC–MS.

Nº	RT	Similarity	Constituent	Molecular Formula	Absolute Area	Relative Area (%)
1	10.119	96	Trimethylsilyl Ester of L-valine	C_11_H_27_NO_2_Si_2_	2,565,504	1.59%
2	15.082	94	Pentanoic acid	C_5_H_10_O_2_	712,900	0.44%
3	16.336	94	Silanol, Phosphoric acid tri-TMS	C_9_H_27_O_4_PSi_3_	28,993,178	17.98%
4	16.492	94	3,7-Dioxa-2,8-disilanonane	C_9_H_24_O_2_Si_2_	3,325,745	2.06%
5	17.056	95	Trimethylsilyl ester of O-trimethylsilyl-L-Threonine	C_10_H_25_NO_3_Si_2_	2,381,219	1.48%
6	17.821	95	Butanedioic acid	C_4_H_6_O_4_	1,288,829	0.80%
7	24.228	94	L-Proline, Pyroglutamic acid di-TMS	C_5_H_7_NO_3_	4,212,701	2.61%
8	24.978	95	Phenylalanine 1TMS	C_12_H_19_NO_2_Si	2,606,598	1.62%
9	31.267	90	Phosphoric acid, α.-Glycer	C_3_H_9_O_6_P	428,872	0.27%
10	32.768	87	Citric acid	C_6_H_8_O_7_	31,491,768	19.53%
11	33.372	90	Glucofuranoside, methyl 2,3,5,6-tetrakis-O-(trimethylsilyl)	C_19_H_46_O_6_Si_4_	6,408,426	3.98%
12	34.591	96	α-D-Galactopyranose	C_6_H_12_O_6_	21,217,490	13.16%
13	36.844	96	β-D-Glucopyranose	C_6_H_12_O_6_	28,402,983	17.62%
14	39.255	91	Myo-inositol	C_6_H_12_O_6_	1,018,404	0.63%
15	41.648	90	Tryptophan 2TMS	C_17_H_28_N_2_O_2_Si_2_	724,774	0.45%
16	50.705	94	Lactose-Octarms	C_36_H_86_O_11_Si_8_	12,470,922	7.74%
17	52.994	92	Maltose-Octarms	C_36_H_86_O_11_Si_8_	12,963,518	8.04%

## Data Availability

The raw data supporting the conclusions of this article will be made available by the authors on request.
